# Nutritional status, health literacy, and lifestyle factors associated with health-related quality of life in patients with decompensated cirrhosis: a cross-sectional study

**DOI:** 10.3389/fpubh.2026.1891516

**Published:** 2026-10-05

**Authors:** Yan Liu, Shuyue He, Tianyu He, Xingyu Liu, Runzhi Zhang, Xin Liu, Tingjuan Huang, Junjie Ren

**Affiliations:** 1 Department of Gastroenterology and Hepatology, The First Hospital of Shanxi Medical University, Taiyuan, China; 2 School of Humanities and Social Sciences, Shanxi Medical University, Taiyuan, China; 3 Department of Gastroenterology and Hepatology, Third Hospital of Shanxi Medical University, Shanxi Bethune Hospital, Shanxi Academy of Medical Sciences, Taiyuan, China; 4 Shanxi Key Laboratory of Metabolic-Immune Homeostasis and Drug Innovation, Taiyuan, Shanxi, China

**Keywords:** health literacy, health management, nutritional status, patients with liver cirrhosis, quality of life

## Abstract

**Background:**

Liver cirrhosis is one of the leading causes of liver-related mortality worldwide. The disease typically progresses from an initially asymptomatic, compensated phase to decompensation, which adversely affects patients’ health-related quality of life (HRQoL). Identifying the factors that influence HRQoL and implementing effective interventions are essential for improving patient outcomes. Based on the health ecology theory, this study focuses on health literacy and nutritional status, with the aim of enhancing patients’ quality of survival through targeted interventions.

**Methods:**

A cross-sectional design was employed, using convenience sampling to recruit 202 patients with decompensated cirrhosis. Data collection included demographic information, biochemical indicators, and scores on health literacy, nutritional status, and HRQoL. Using IBM SPSS Statistics version 27, correlation analysis and multiple linear regression were conducted to primarily explore the associations of nutritional status, health literacy, and lifestyle factors with HRQoL.

**Results:**

Correlation analysis showed that health literacy, nutritional status, sleep quality, and physical activity were significantly associated with patients’ quality of life. Multiple linear regression revealed that nutritional status was independently associated with the HRQoL; notably, the model’s explanatory power for HRQoL significantly increased (*R* Square increment) when health literacy was introduced, indicating practical significance. Furthermore, regression analyses showed that communication ability (*B* = −0.177, *p* = 0.007), walking (*B* = −1.589, *p* = 0.007), and aerobic exercise (*B* = −1.739, *p* = 0.017) as significant predictors of the physical dimension. Poor sleep significantly predicted both dimensions (physical: *B* = 1.193, *p* = 0.032; psychological: *B* = 0.532, *p* = 0.001), while information acquisition ability (*B* = −0.028, *p* = 0.021) specifically predicted the psychological dimension.

**Conclusion:**

This study demonstrated that both health literacy and nutritional status were significantly associated with the HRQoL among patients with decompensated cirrhosis. These findings may inform the design of targeted interventions to enhance health literacy and nutritional status, which may be beneficial for promoting self-management and improving clinical outcomes. Furthermore, they support the development of individualized management strategies.

## Introduction

Cirrhosis is the main factor leading to the incidence and mortality of patients with chronic liver disease worldwide. Each year, liver diseases result in over 2 million deaths, accounting for 4% of total global mortality (1). The progression of cirrhosis typically begins with an asymptomatic compensated phase, followed by decompensation characterized by clinical symptoms such as ascites, bleeding, hepatic encephalopathy, and jaundice (2). Complications arising from the transition from compensated to decompensated stages often lead to hospitalization, reducing quality of life, and increasing mortality (3). Currently, the etiologies of cirrhosis is changing. In Europe, non-alcoholic fatty liver disease (NAFLD) is rising while alcoholic cirrhosis appears to be declining; however, viral hepatitis remains the primary cause in the Eastern Mediterranean and Western Pacific regions (4). Regardless of etiology, patients generally face a decline in health-related quality of life (HRQoL), which is particularly pronounced during the decompensated stage (5, 6).

The disease’s impact on patients’ HRQoL is a crucial health indicator. It focuses on their physical condition and functional status (7). In all studies assessing these factors, depression, poor sleep, and muscle spasms have been found to affect HRQoL (8). Concurrently, alcohol consumption, smoking, malnutrition, along with common comorbidities like chronic obstructive pulmonary disease, musculoskeletal disorders, and psychiatric conditions, contribute to a decline in HRQoL among patients with alcoholic liver disease (9–11). Notably, malnutrition serves as a significant risk factor for sarcopenia, which has been proven to be a favorable predictor for the outcomes of cirrhosis (12). Identifying the factors influencing HRQoL in patients with cirrhosis is crucial for improving patient outcomes and optimizing treatment strategies.

Health literacy also influences the health outcomes of patients with chronic diseases. It is generally defined as an individual’s cognitive and communicative abilities to access, understand, and use information to promote and maintain good health (13). Studies have categorized health literacy into three levels: functional health literacy refers to basic reading and writing skills necessary for daily life operations; communicative health literacy involves recognition and literacy skills used to extract information and gain meaning; critical health literacy represents higher cognitive skills utilized to better control daily life and situations (14, 15). As a key component of effective self-care and disease management (16), improving population-level health literacy serves as a favorable factor in promoting the large-scale treatment of hepatitis B in China (17). A comparative analysis of gastrointestinal disease patients revealed that patients with cirrhosis scored lowest in health literacy, particularly showing significant deficiencies in information acquisition, interaction with healthcare providers, and evaluation of health information (18). Poor liver function status and cirrhosis complications are associated with lower health literacy (19). Regardless of the severity of liver disease, patients with low health literacy are more than three times as likely to experience severe physical frailty compared to others (20) and tend to have higher rates of emergency re-visits (21). Enhancing health literacy has a positive impact on both the healthcare system and individual health outcomes.

Research focusing on the impact of health literacy on the HRQoL among patients with cirrhosis remains relatively scarce. This study effectively fills the research gap regarding the potential role of health literacy in the health management and interdisciplinary comprehensive clinical management of patients with decompensated cirrhosis, an area for which the current evidence base remains relatively weak.

This study identifies health literacy and nutritional status as primary independent variables to evaluate their influence on HRQoL in these patients. The selection of these variables is theoretically grounded in the Health Ecology Model. Health Ecology Model posits that individual health outcomes are shaped by a complex interplay of personal characteristics, behavioral factors, social networks, environmental contexts, and policy environments (22). Implementing targeted interventions at both the individual and structural levels to enhance health literacy is associated with patient outcomes.

## Methods

### Sample size determination and inclusion/exclusion criteria

This study employed a cross-sectional design, utilizing convenience sampling to consecutively enroll 202 patients with cirrhosis admitted to the Department of Gastroenterology at the First Hospital of Shanxi Medical University between July 2024 and August 2025. A total of 202 valid responses were obtained. Sample size estimation was performed using G*Power software. For the multiple linear regression analysis, we specified 10 predictors, a medium effect size (*f*^2^ = 0.15), a significance level (*α*) of 0.05, and a statistical power (1–*β*) of 0.80. The calculated minimum required sample size was 118 cases. Our study enrolled 202 patients, exceeding this threshold and thus providing sufficient power to detect the assumed effect. Moreover, a sensitivity analysis based on the actual sample size (*N* = 202) revealed that the study had 80% power to detect a minimum effect size as low as *f*^2^ = 0.08 (*R*^2^ ≈ 0.074), which is smaller than the conventional medium effect, further substantiating the adequacy of our sample size. Inclusion criteria comprised: (1) voluntary signing of an informed consent form; (2) age ≥16 years; (3) clinical diagnosis of cirrhosis confirmed by clinical manifestations, imaging examinations, biochemical indicators, and liver biopsy; (4) consumption of regular diet without receiving nutritional support; (5) hospitalization duration exceeding 2 days. Exclusion criteria included: (1) inability to orally ingest food; (2) prior or current receipt of nutritional support therapy; (3) severe disability or dementia preventing cooperation with assessment or effective communication.

### Instruments and measurements

#### Demographic and anthropometric information

Demographic data include gender, age, educational level, occupation type, living style, household registration, smoking and drinking habits, etc. Additionally, researchers utilized a dynamometer, tape measure, and skinfold caliper to measure participants’ body mass index (BMI), mid-upper arm circumference (MUAC), grip strength, and triceps skinfold thickness.

#### Laboratory indicators

On the second day of admission, fasting blood samples were collected in the morning to assess laboratory indicators, including platelet count, hemoglobin (Hb), albumin (Alb), Alanine Aminotransferase (ALT), Aspartate Aminotransferase (AST), bilirubin (Bil), prothrombin time (PT), prothrombin activity, international normalized ratio (INR), D-dimer, urea, creatinine (Cr), cholesterol, triglycerides, low-density lipoprotein (LDL), blood glucose, blood ammonia, and serum sodium.

#### Health literacy management scale(HeLMS)

The HeLMS scale was developed by Jordan et al. (23). The Chinese translated version comprises 24 items, divided into four dimensions: Information Acquisition Ability (9 items), Communication and Interaction Ability (9 items), Willingness to Improve Health (4 items), and Willingness for Economic Support (2 items). Willingness for Economic Support is defined as the extent to which patients with cirrhosis are willing to invest personal resources (e.g., energy and time) for health benefits and to make proactive lifestyle modifications to improve disease outcomes. It utilizes a 5-point Likert scale, ranging from “Completely impossible” (1 point) to “Not difficult at all” (5 points). The total score ranges from 24 to 120; higher scores indicate better health literacy levels. The mean (SD) health literacy of patients was 90.94 (19.27). The Cronbach’s α coefficient for the entire scale is 0.959, while the Cronbach’s α coefficients for each dimension are 0.95, 0.90, 0.94, and 0.93.

#### Subjective global assessment (SGA)

The SGA scale encompasses eight aspects, including weight changes over the past 6 months, dietary changes, gastrointestinal symptoms, activity level, stress response, muscle wasting, triceps skinfold thickness, and ankle edema. It categorizes nutritional status into three grades: A (Well-nourished), B (Mild-to-moderate malnutrition), and C (Severe malnutrition). The classification criteria are as follows (24):

Grade C (Severe): At least 5 out of 8 items rated as Grade C.

Grade B (Moderate): If Grade C < 5 but Grade B + C ≥ 5.

Grade A (Well-nourished): If Grade B + C < 5.

In this study, there were 114 patients in Grade A (well-nourished), 79 in Grade B (mild-to-moderate), and 9 in Grade C (severe). Due to the small sample size of Grade C, to better demonstrate differences in various indicators among patients with different nutritional statuses, we combined the Grade C group with the Grade B group to form the Malnutrition Group = 2, while the Grade A group served as the normal nutrition group = 1. Moreover, the three-category sensitivity analysis yielded conclusions that were fully consistent with those from the merged binary model, indicating that our merging strategy did not introduce substantial bias into the primary findings.

#### Health-related quality of life(EQ-5D-5L)

The EQ-5D-5L questionnaire is utilized to measure and evaluate HRQoL (25). It consists of five dimensions: mobility, self-care, usual activities, pain or discomfort, and anxiety or depression. Each dimension is rated on a five-point scale ranging from “no problems” to “extreme problems.” The total score ranges from 5 to 25 points; higher scores indicate poorer HRQoL. The mean (SD) HRQoL of patients was10.02 (4.89). The overall Cronbach’s α coefficient for the scale is 0.88.

### Statistical analysis

Data analysis was conducted using IBM SPSS Statistics version 27. Cronbach’s alpha coefficients were calculated to assess internal consistency of the scales, and the health-related quality of life and health literacy data were approximately normally distributed. Descriptive statistics were presented as mean, standard deviation, frequency, and percentage. Independent samples t-tests and chi-square tests were utilized to compare differences in HRQoL, health literacy, and nutritional status across various demographic variables and biochemical indicators. Pearson correlation coefficients were used to examine the correlations among HRQoL, health literacy, and lifestyle factors. Furthermore, multiple linear regression models were used to examine the independent associations of health literacy with HRQoL. Additionally, linear regression analyses were performed to compare the associations of variables with the physical and mental dimensions of HRQoL. Independent variables were entered using the Enter method. Due to the low proportion of missing data, no imputation was performed for missing values in the regression analysis; instead, the default SPSS listwise deletion procedure was adopted, meaning that only cases with complete data for all variables involved in the regression model were included. The specific sample size for each regression model is detailed in the results tables.

## Results

### Description statistics of variables

The results indicated that patients aged 60 years and younger generally exhibited better HRQoL and higher levels of health literacy, along with improved nutritional status. Furthermore, patients with urban household registration and higher educational attainment demonstrated significantly higher scores in health literacy. Non-agricultural laborers showed better HRQoL and higher health literacy scores. Patients with abnormal albumin levels presented poorer HRQoL, and those with abnormal total bilirubin levels tended to have relatively worse nutritional status (see Table 1).

**Table 1:** Distribution of health-related quality of life, health literacy and nutritional status among patients according to demographic characteristics (*n* = 202).

Variable		HRQoL	Health literacy	Nutritional status	
				Well-nourished	Malnourished
Gender	male	9.85 ± 4.91	92.40 ± 18.31	63 (55.3%)	46 (52.3%)
	female	10.21 ± 4.88	89.23 ± 20.21	51 (44.7%)	42 (47.7%)
	*t*/Chi-square	−0.51	1.16	0.18	
Age group	≤60	8.66 ± 4.26	96.97 ± 17.33	67 (58.8%)	39 (44.3%)
	61~	11.47 ± 5.15	84.53 ± 19.28	47 (41.2%)	49 (55.7%)
	*t*/Chi-square	−4.21***	4.80***	4.16*	
Household registration	Urban	10.02 ± 4.66	94.20 ± 18.54	59 (51.8%)	52 (59.1%)
	Rural	10.01 ± 5.18	86.96 ± 19.50	55 (48.2%)	36 (40.9%)
	*t*/Chi-square	0.01	2.69*	1.08	
Educational level	Vocational School or Below	10.38 ± 5.01	86.92 ± 19.70	84 (73.7%)	64 (72.7%)
	Senior High School or Above	9.04 ± 4.46	101.81 ± 12.98	30 (26.3%)	24 (27.3%)
	*t*/Chi-square	1.73	−5.16***	0.02	
Employment status	Agricultural	10.44 ± 5.08	86.50 ± 19.70	83 (72.8%)	64 (72.7%)
	Non-agricultural	8.90 ± 4.19	102.64 ± 11.88	31 (27.2%)	24 (27.3%)
	*t*/Chi-square	2.02*	−5.69***	0.00	
Hb	Normal	8.88 ± 4.28	94.18 ± 19.46	32 (28.1%)	19 (21.6%)
	Abnormal	10.41 ± 5.03	90.01 ± 19.15	82 (71.9%)	69 (78.4%)
	*t*/Chi-square	−1.92	1.32	1.11	
Alb	Normal	7.75 ± 2.75	99.00 ± 16.08	14 (12.3%)	8 (9.1%)
	Abnormal	10.30 ± 5.03	90.07 ± 19.46	100 (87.7%)	80 (90.9%)
	*t*/Chi-square	−2.22**	1.98	0.52	
Cr	Normal	10.13 ± 5.02	90.86 ± 18.85	82 (71.9%)	66 (75.0%)
	Abnormal	9.84 ± 4.58	91.29 ± 20.70	32 (28.1%)	22 (25.0%)
	t/Chi-square	0.36	−0.14	0.24	
Bil	Normal	9.96 ± 4.91	91.58 ± 18.85	27 (23.7%)	32 (36.4%)
	Abnormal	10.14 ± 4.90	90.21 ± 20.17	87 (76.3%)	56 (63.6%)
	*t*/Chi-square	0.23	−0.45	3.87*	
ALT	Normal	9.98 ± 4.75	91.47 ± 18.86	85 (74.6%)	69 (78.4%)
	Abnormal	10.42 ± 5.45	89.42 ± 20.94	29 (25.4%)	19 (21.6%)
	*t*/Chi-square	−0.53	0.62	0.41	
AST	Normal	10.02 ± 4.98	90.83 ± 19.09	66 (57.9%)	44 (50.0%)
	Abnormal	10.15 ± 4.85	91.20 ± 19.71	48 (42.1%)	44 (50.0%)
	*t*/Chi-square	−0.20	−0.13	1.25	

Hb, hemoglobin; Alb, albumin; Cr, creatinin; Bil, bilirubin; ALT, Alanine Aminotransferase; AST, Aspartate Aminotransferase.

*p < 0.05, **p < 0.01, ***p < 0.001.

### Correlation analysis of main variables

The correlation analysis of the main variables revealed that each dimension of health literacy is significantly negatively correlated with both the physical and psychological aspects of HRQoL (*p* < 0.01). Specifically, higher levels of information acquisition ability, communication interaction ability, willingness to improve health, and willingness to provide economic support are associated with better physical and psychological status in patients. Nutritional status showed a positive correlation with both physical and psychological status (*r* = 0.25, *p* < 0.01; *r* = 0.18, *p* < 0.05), indicating that patients with malnutrition tended to have poorer physical and psychological conditions. Physical activity status was negatively correlated with both physical and psychological status (*r* = −0.21, *p* < 0.01; *r* = −0.19, *p* < 0.05), suggesting that lower intensity of physical activity was linked to worse physical and psychological well-being. Sleep status showed a positive correlation with both physical and psychological status (*r* = 0.23, *p* < 0.01; *r* = 0.26, *p* < 0.05), indicating that better sleep quality is associated with better physical and psychological conditions (see Table 2).

**Table 2:** Correlation matrix of variables (*n* = 200).

Variable	1	2	3	4	5	6	7	8	9	10
HRQoL										
1. HRQoL-Physical Dimension	1									
2. HRQoL - Psychological Dimension	0.44**	1								
Health literacy										
3. Information acquisition ability	−0.45**	−0.35**	1							
4. Communication and interaction ability	−0.45**	−0.29**	0.67**	1						
5. Willingness to improve health	−0.34**	−0.23**	0.58**	0.77**	1					
6. Willingness for economic support	−0.32**	−0.33**	0.58**	0.57**	0.58**	1				
7. Nutritional status	0.25**	0.18*	−0.10	−0.05	−0.03	−0.07	1			
Lifestyle										
8. Alcohol consumption	−0.14*	−0.06	0.22**	0.02	0.09	0.15*	−0.04	1		
9. Physical activity	−0.21**	−0.19**	0.12	0.23**	0.22**	0.24**	−0.03	−0.07	1	
10. Sleep status	0.23**	0.26**	−0.14*	−0.15*	−0.01	−0.07	0.20**	−0.18	−0.01	1

HRQoL:health-related quality of life.

*p < 0.05, **p < 0.01, ***p < 0.001.

The physical activity data were missing for 2 patients.

### Hierarchical multiple linear regression analysis of HRQoL

Hierarchical multiple linear regression was performed with HRQoL as the dependent variable. Variable selection combined a theory-driven approach with hierarchical entry. Candidate variables (demographics, disease severity, lifestyle, nutritional status, and health literacy) were predefined based on the literature. Hierarchical regression was used to force-enter variable blocks in three sequential models to assess the independent contribution of each domain to HRQoL. In Model 1, age, physical activity (both walking and aerobic exercise vs. sedentary), and poor sleep (vs. good sleep) significantly affected HRQoL (all *p* < 0.01), collectively explaining 26.5% of the variance (*R*^2^ = 0.265). When nutritional status was introduced in Model 2, the R^2^ slightly increased to 0.285, with a significant R^2^ change of 2.0%, indicating an independent contribution of nutritional status to HRQoL (*B* = 1.429, *p* = 0.027). Patients with malnutrition exhibited worse quality of life. The most substantial improvement occurred in Model 3 with the addition of health literacy. The *R*^2^ increased to 0.392, representing a marked *R*^2^ change of 10.7% (*p* < 0.001), suggesting that the increase in the model’s explanatory power was independently associated with health literacy, higher health literacy was significantly associated with lower HRQoL scores, indicating better health status. The Durbin-Watson statistic was 2.005, confirming no significant autocorrelation in the residuals and validating the robustness of the final model (see Table 3). Multicollinearity was assessed by VIF, and all values (1.070–1.537) were below 2, indicating no significant collinearity issues.

**Table 3:** Hierarchical multiple linear regression analysis of HRQoL (*n* = 191).

Variable	Model 1			Model 2			Model 3		
	*B*	*β*	*t*	*B*	*β*	*t*	*B*	*β*	*t*
Constant	1.749 (2.007, 5.506)	—	0.919	0.176 (0.931–3.794)	—	0.087	12.479 (6.810, 18.148)	—	4.344^***^
Age	0.151 (0.096, 0.207)	0.359	5.379^***^	0.141 (0.068, 0.197)	0.335	5.010^***^	0.082 (0.027, 0.137)	0.195	2.922^**^
MELD	−0.024 (−0.172, 0.125)	−0.024	−0.313	−0.015 (−0.162, 0.133)	−0.015	−0.195	−0.080 (−0.218, 0.058)	−0.083	−1.146
Child-Pugh B (vs A)	0.687 (−0.792, 2.166)	0.069	0.917	0.722 (−0.742, 2.185)	0.073	0.973	1.274 (−0.092, 2.641)	0.128	1.840
Child-Pugh C (vs A)	−0.678 (−4.178, 2.823)	−0.028	−0.382	−0.559 (−4.023, 2.906)	−0.023	−0.318	0.342 (−2.876, 3.561)	0.014	0.210
Sleep-moderate (vs good)	1.534 (−0.630,3.698)	0.094	1.398	1.367 (−0.779,3.513)	0.084	1.0.257	0.641 (−1.359,2.642)	0.039	0.632
Sleep-poor (vs good)	2.498 (1.161, 3.835)	0.247	3.686^***^	2.276 (0.939, 3.614)	0.225	3.358^***^	1.900 (0.657, 3.144)	0.188	3.015^**^
Pa-walking (vs sedentary)	−2.867 (−4.305, −1.429)	−0.275	−3.934^***^	−2.701 (−4.131, −1.271)	−0.259	−3.727^***^	−1.986 (−3.332, −0.640)	−0.191	−2.911^**^
Pa-aerobic (vs sedentary)	−3.855 (−5.595, −2.115)	−0.305	−4.372^***^	−3.777 (−5.500, −2.054)	−0.299	−4.326^***^	−2.201 (−3.888, −0.515)	−0.174	−2.576^*^
Nutritional status				1.429 (0.162, 2.695)	0.145	2.226^*^	1.500 (0.328, 2.671)	0.152	2.527^**^
Health literacy							−0.096 (−0.129, −0.062)	−0.373	−5.619^***^
*R* ^2^	0.265			0.285			0.392		
Adj. *R*^2^	0.233			0.249			0.358		
Durbin-Watson	—			—			2.005		
*F*	8.175^***^			7.975 ^***^			11.554^***^		

*p < 0.05, **p < 0.01, ***p < 0.001.

MELD, Model for End-Stage Liver Disease.

Child-Pugh classification was entered into the regression model as dummy variables, with class A as the reference group. Two dummy variables were created: class B (1 = class B, 0 = otherwise) and class C (1 = class C, 0 = otherwise).

Sleep status was entered into the regression model as dummy variables, with “good” as the reference group. Two dummy variables were created: “moderate” (1 = moderate, 0 = otherwise) and “poor” (1 = poor, 0 = otherwise).

Physical activity (Pa) was entered into the regression model as dummy variables with “sedentary” as the reference group. Two dummy variables were created: “walking” (1 = walking, 0 = otherwise) and “aerobic exercise” (1 = aerobic exercise, 0 = otherwise).

11 patients were follow-up outpatients after discharge, and their MELD and Child scores were missing.

### Regression analysis and comparison of physical and psychological dimensions of HRQoL

The regression analyses revealed distinct patterns between the physical and psychological dimensions of HRQoL. For the physical dimension (Table 4), the model explained 36.7% of the variance (*R*^2^ = 0.367, *F* = 10.968, *p* < 0.001). Significant predictors included age (B = 0.085, *p* < 0.001), poor sleep (*B* = 1.193, *p* = 0.032), walking (B = -1.589, *p* = 0.007), aerobic exercise (*B* = −1.739, *p* = 0.017), nutritional status (*B* = 1.141, *p* = 0.028), and communication ability (*B* = −0.177, *p* = 0.007). For the psychological dimension (Table 5), the model explained 23.3% of the variance (*R*^2^ = 0.233, *F* = 5.741, *p* < 0.001), with poor sleep (*B* = 0.532, *p* = 0.001) and information acquisition ability (*B* = −0.028, *p* = 0.021) as significant predictors. Sleep quality emerged as a shared predictor across both dimensions. However, the physical dimension was influenced by a broader range of factors, whereas the psychological dimension was more specifically associated with sleep and information acquisition. These findings highlight the need for comprehensive strategies targeting multiple domains to improve overall HRQoL.

**Table 4:** Multiple linear regression analysis of HRQoL-Physical Dimension (*n* = 200).

Variable	*B*	*β*	*t*	Sig.	95% Confidence Interval		VIF
					LL	UL	
(Constant)	8.343	—	3.520	<0.001	3.668	13.018	—
Age	0.089	0.246	3.517	<0.001	0.039	0.138	1.462
Sleep-moderate (vs good)	−0.079	−0.006	−0.090	0.928	−1.814	1.655	1.195
Sleep-poor (vs good)	1.193	0.134	2.157	0.032	0.102	2.284	1.160
Pa-walking (vs sedentary)	−1.589	−0.175	−2.703	0.007	−2.748	−0.429	1.257
Pa-aerobic (vs sedentary)	−1.739	−0.157	−2.405	0.017	−3.166	−0.313	1.271
Nutritional status	1.141	0.133	2.207	0.028	0.121	2.161	1.084
Information acquisition ability	−0.058	−0.127	−1.412	0.160	−0.138	0.023	2.412
Communication and interaction ability	−0.177	−0.286	−2.746	0.007	−0.305	−0.050	3.233
Willingness to improve health	0.092	0.079	0.830	0.407	−0.127	0.311	2.705
Willingness for economic support	−0.081	−0.043	−0.530	0.597	−0.381	0.220	1.957
*R* ^2^	0.367						
Adj. *R*^2^	0.334						
Durbin-Watson	2.024						
*F*	10.968 (*p* < 0.001)						

The physical activity data were missing for 2 patients.

**Table 5:** Multiple linear regression analysis of HRQoL-Psychological Dimension (*n* = 200).

Variable	*B*	*β*	*t*	Sig.	95% Confidence Interval		VIF
					LL	UL	
(Constant)	3.609	—	5.133	<0.001	2.222	4.996	—
Age	−0.009	−0.093	−1.211	0.227	−0.024	0.006	1.462
Sleep-moderate (vs good)	0.299	0.080	1.146	0.253	−0.215	0.813	1.195
Sleep-poor (vs good)	0.532	0.222	3.241	0.001	0.208	0.855	1.160
Pa-walking (vs sedentary)	−0.015	0.006	−0.084	0.933	−0.359	0.329	1.257
Pa-aerobic (vs sedentary)	−0.407	−0.136	−1.898	0.051	−0.830	0.016	1.271
Nutritional status	0.280	0.121	1.828	0.069	−0.022	0.583	1.084
Information acquisition ability	−0.028	−0.230	−2.324	0.021	−0.052	−0.004	2.412
Communication and interaction ability	−0.003	−0.019	−0.167	0.867	−0.041	0.035	3.233
Willingness to improve health	0.008	0.026	0.249	0.804	−0.057	0.073	2.705
Willingness for economic support	−0.076	−0.150	−1.688	0.093	−0.166	0.013	1.957
*R* ^2^	0.233						
Adj. *R*^2^	0.192						
Durbin-Watson	1.803						
*F*	5.741 (*p* < 0.001)						

The physical activity data were missing for 2 patients.

## Discussion

This study analyzed the relationships among health literacy, nutritional status, and HRQoL in patients with decompensated cirrhosis. The results indicated that both health literacy and nutritional status were significantly correlated with HRQoL in this population. Overall, higher health literacy was significantly associated with better HRQoL. Further stratified analysis revealed that communication skills influence the physical health of cirrhosis patients, while information acquisition ability affects their mental health. A prospective study by Ankit V Patel et al. showed that, under similar severity of liver disease, patients with higher cognitive reserve performed better in terms of HRQoL (26). Hidden hepatic encephalopathy and low educational level are common risk factors for low health literacy (27). Interventions aimed at improving health literacy in patients with cirrhosis are becoming increasingly diverse, seeking to enhance self-management capabilities through education, technology, and behavioral support. Research by Jessica Au et al. demonstrated that digital health interventions have a unique advantage in improving health literacy among patients with decompensated cirrhosis; tneir conversational interactions can simultaneously improve patients’ mental health status (28). Delivering educational videos via mobile service platforms to enhance patient health literacy is crucial for the sustainable prevention of lifestyle-related diseases (29). Meanwhile, healthcare providers, public health systems, and social support play important roles in promoting health literacy, influencing people’s ability to access and utilize healthcare services (30, 31). For instance, specialized liver nurses play a key role in helping cirrhosis patients identify and appropriately manage symptoms, thereby improving their medical experience (32).

Patients with malnutrition had significantly worse HRQoL, and this association was more pronounced in the physical dimension. Malnutrition is a common characteristic and complication in cirrhosis patients, associated with poor outcomes (33–36). The prevalence of malnutrition or sarcopenia in cirrhosis patients ranges from 23 to 60% (37). However, the prevalence varies depending on assessment method and disease severity (38). Sarcopenia, one of the core manifestations of malnutrition, serves as an independent predictor of incidence and mortality (39–41). Optimizing nutritional status can improve morbidity, mortality, and overall outcomes (42). Studies suggest that appropriate intensity aerobic exercise and resistance training (43), as well as exercise interventions (44), can be implemented. Developing self-management plans focused on exercise and nutrition can effectively improve the quality of life for cirrhosis patients (45).

The study results indicated that physical activity was associated with patients’ physical health. A review analysis showed that exercise and exercise interventions can improve frailty status, quality of life, and patient outcomes in liver disease patients (46–48). Furthermore, a comprehensive study linking a diet-exercise pattern with cirrhosis found that adjusting diet and increasing physical activity, while simultaneously controlling blood pressure and glucose levels, can better prevent the onset of cirrhosis (49). However, the provision of exercise programs must reflect individualization (50).

Furthermore, sleep status is significantly associated with the HRQoL of patients with liver cirrhosis, and this association is evident in both the physical and psychological dimensions. A prospective study was the first to combine actigraphy with subjective assessments to evaluate sleep disturbances in Romanian patients with chronic liver disease, demonstrating a link between sleep disorders and decreased HRQoL (51). Sleep disorders, social functions are associated with chronic pain in patients with liver cirrhosis (52). Additionally, symptoms of depression and anxiety are prevalent among these patients (53). A multicenter cross-sectional study revealed that depression partially mediates the relationship between insomnia and quality of life in patients with advanced hepatocellular carcinoma (54). Therefore, it is crucial to appropriately identify and manage these symptoms to improve the overall well-being of this population.

In conclusion, the clinical management of liver cirrhosis should adopt a patient-centered approach emphasizing multidisciplinary collaboration (55, 56). In the era of digital intelligence, through technological empowerment, diverse educational content related to disease cognition and self-management is provided to patients, thereby overall enhancing their health literacy levels. A comprehensive management plan developed by physicians, nurses, social workers, dietitians, and mental health professionals may be beneficial for improving patients’ quality of life and outcomes. Future research should investigate the long-term impact of these interventions on clinical outcomes.

## Limitations

This study employs a single-center cross-sectional design, and the use of convenience sampling may introduce selection bias. Data were collected through face-to-face interviews in accordance with the established inclusion and exclusion criteria. Cross-sectional studies cannot infer causal relationships between variables, and the impact of comorbidities has not been explored. These limitations provide a basis for future research. Further studies are needed to validate the mechanisms of health literacy in disease management and to explore the complexities of multimorbidity. Meanwhile, nutritional status in patients with decompensated cirrhosis is an important clinical concern. In subsequent specialized studies on nutritional problems, measurement tools should be comprehensively utilized to enhance the comprehensiveness of the assessment.

## Conclusion

Higher levels of health literacy, nutritional status, and sleep quality in patients with liver cirrhosis are associated with better HRQoL. While the improvement of health literacy requires not only patients’ active engagement in learning and changes in disease management cognition, but also necessitates the broader healthcare system to provide adequate social support.

## Data Availability

The raw data supporting the conclusions of this article will be made available by the authors, without undue reservation.
