# Supplementary material for: Nutritional status, health literacy, and lifestyle factors associated with health-related quality of life in patients with decompensated cirrhosis: a cross-sectional study
Source: Front Public Health. 2026 Oct 5;14:1891516. doi: 10.3389/fpubh.2026.1891516 (PMC13677692; doi:10.3389/fpubh.2026.1891516)
Supplement: Supplementary file 1 [file Supplementary_file_1.pdf]

The SGA classification

Regarding the rationale for the original merging scheme:

Our initial decision to merge SGA classes B and C was based primarily on two considerations: (1) Clinical perspective — in clinical practice, both SGA class B (moderate malnutrition) and class C (severe malnutrition) are generally regarded as positive indicators for "nutritional intervention." Compared with class A (well-nourished), they share a consistent clinical decision-making threshold; therefore, merging them into a single "malnourished" group (well-nourished vs. malnourished) better aligns with actual clinical operations. (2) Statistical perspective — only 9 patients (4.5% of the total sample) were classified as class C. If treated as an independent group in the multiple regression model, the parameter estimates would be highly unstable (with markedly inflated standard errors), potentially leading to model overfitting and insufficient statistical power.

Sensitivity analysis:

We have re-run the multiple linear regression model with SGA as a three-category variable (classes A, B, and C) using dummy variables (with class A as the reference). The results are presented in the table below(**A portion has been extracted**). In Model 2, using class A as the reference, the regression coefficient for class B was β = .126 (P = .051), and for class C was β = .129 (P = .045). In Model 3, with class A as the reference, the coefficient for class B was β = .140 (P = .023), and for class C was β = .104 (P = .088). The regression coefficient for health literacy was β = −.368 (P < .001). The three-category model yielded conclusions that were fully consistent with the original binary model, indicating that our merging strategy did not lead to any substantial bias in the primary findings.

Model		B	Std.Error	β	t	*P*	95%Confidence Interval	
							LL	UL
3	Constant	13.833	2.818		4.909	.000	8.273	19.394
	Age	.082	.028	.195	2.925	.004	.027	.138
	Meld	-.079	.070	-.081	-1.120	.264	-.217	.060
	Child-Pugh B（vs A）	1.248	.694	.126	1.797	.074	-.122	2.618
	Child-Pugh C（vs A）	.361	1.633	.015	.221	.826	-2.862	3.584
	Sleep-moderate(vs good)	.700	1.018	.043	.688	.493	-1.309	2.709
	Sleep-poor(vs good)	1.905	.631	.188	3.018	.003	.659	3.150
	Pa-walking (vs sedentary)	-1.992	.683	-.191	-2.917	.004	-3.340	-.644
	Pa-aerobic (vs sedentary)	-2.163	.857	-.171	-2.524	.012	-3.854	-.472
	SGA-B(vs A)	1.395	.610	.140	2.288	.023	.192	2.598
	SGA-C(vs A)	2.528	1.472	.104	1.717	.088	-.377	5.433
	Health literacy	-.094	.017	-.368	-5.516	.000	-.128	-.061

Based on the above sensitivity analysis, we believe that although the merging strategy entails a modest loss of granularity in statistical terms, we retain the binary classification in the main analysis for the following reasons: (1) the clinical rationale that both classes B and C warrant nutritional intervention; (2) the sensitivity analysis confirms the robustness of the primary conclusions; and (3) the small sample size of class C poses statistical risks if analysed independently. We have therefore presented the three-category sensitivity analysis results as supplementary material, thereby balancing clinical applicability with statistical rigour.
